# Deciphering the Multifaceted Relationship between Oncolytic Viruses and Natural Killer Cells

**DOI:** 10.1155/2012/702839

**Published:** 2011-12-11

**Authors:** Christopher A. Alvarez-Breckenridge, Jianhua Yu, Balveen Kaur, Michael A. Caligiuri, E. Antonio Chiocca

**Affiliations:** ^1^Medical Scientist Training Program, The Ohio State University, Columbus, OH 43210, USA; ^2^Dardinger Laboratory for Neuro-Oncology and Neurosciences, Department of Neurological Surgery, The Ohio State University, Columbus, OH 43210, USA; ^3^The Ohio State University Comprehensive Cancer Center, The Ohio State University, Wiseman Hall, 400 West 12th Avenue, Columbus, OH 43210, USA

## Abstract

Despite active research in virotherapy, this apparently safe modality has not achieved widespread success. The immune response to viral infection appears to be an essential factor that determines the efficacy of oncolytic viral therapy. The challenge is determining whether the viral-elicited immune response is a hindrance or a tool for viral treatment. NK cells are a key component of innate immunity that mediates antiviral immunity while also coordinating tumor clearance. Various reports have suggested that the NK response to oncolytic viral therapy is a critical factor in premature viral clearance while also mediating downstream antitumor immunity. As a result, particular attention should be given to the NK cell response to various oncolytic viral vectors and how their antiviral properties can be suppressed while maintaining tumor clearance. In this review we discuss the current literature on the NK response to oncolytic viral infection and how future studies clarify this intricate response.

## 1. Introduction

The field of oncolytic viral therapy is currently at a crossroads. With over twenty years of attention directed towards oncolytic viruses (OV), clinical trials have been encouraging, but have left investigators with the task of identifying barriers that can be circumvented to achieve more successful virotherapy. Some of the most prevalent obstacles include the antiviral host response to OV, the angiogenic response to viral infection, extracellular barriers to viral spread, and inefficient/nonspecific receptor-ligand interactions on target cells [1]. Interestingly, various groups have also demonstrated that an inability to achieve adequate antitumor immunity also represents a significant barrier to tumor clearance [2]. In order to optimize virotherapy for clinical success, the relevance of these barriers, along with the conflicting roles of antiviral and antitumor immunity, must be clarified. While various groups have studied the host response to OV, the natural killer (NK) cell response to various oncolytic viruses has been less thoroughly investigated. 

**Figure 1:**
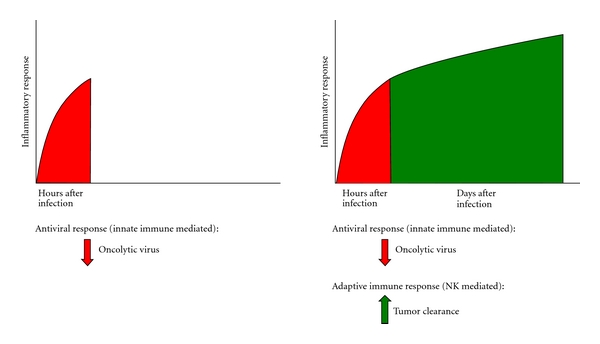
The immune reaction to oncolytic viral infection is two-phased response. Within hours after infection, the innate immune response consisting of NK cells, macrophages, and neutrophils is recruited to the site of infection and mediates initial viral clearance. Following this response to infection, innate immune mediators, particularly NK cells, mediate the downstream adaptive immune response that is a critical antitumor mediator. In order to reconcile this biphasic response, initial immune suppression targeting NK cells may be required initially after viral infection followed by a period of immune stimulation to elicit antitumor immunity.

In order to appreciate both the current literature surrounding the NK response to OV therapy and understand how these cells can be targeted in future studies, it is essential to understand the role of these cells in viral clearance and tumor immunology. Interestingly, profound human NK cell deficiencies have led to overwhelming herpes viral infections, supporting the notion that this innate immune effector cell has specific recognition of, and control over, viral infection [3–5]. Additionally, multiple reports have associated NK cell levels with tumor regression [6–9]. Taken together, these findings highlight potentially conflicting roles for NK cells in oncolytic virotherapy. On the one hand, the antiviral properties of these cells may be detrimental to viral propagation and viral mediated tumor clearance. Conversely, an activated NK response following OV infection of tumors may stimulate NK-mediated antitumor immunity (Figure 1). While most studies to date have focused on the dichotomous nature of the NK response, it is likely that a more nuanced approach will be needed in which the antiviral response to infection is initially suppressed while antitumor immunity is selectively stimulated.

Investigators frequently attempt to correlate the success of their oncolytic viral therapy with immune cell infiltration following infection. Using this metric, NK cells have been highlighted as a relevant factor in response to OV infection. However, significantly less attention has been directed towards the nature and relevance of this viral-induced NK response. For example, what role do NK cells have in recruiting activated macrophages following OV therapy? Does OV administration induce a different NK activation profile compared to infection with its wild-type counterpart? Does OV infection of tumor lead to the preferential NK-mediated clearance of these virally infected cells compared to uninfected tumor and therefore impeded viral oncolysis? Are there discrepancies between activated NK cells that are recruited in mice bearing xenograft tumors versus syngeneic tumors? Lastly, is it possible to temporarily pharmacologically modulate the NK immune response to OV-infected cells in order to enhance OV therapeutic efficacy? These are just a few of the questions that should be considered as investigators move beyond just determining that these cells are recruited following infection. In this review, we will discuss the nature of the host response to OV infection, highlight the clinical relevance of NK cells in antiviral defense, consider the literature surrounding the NK response to OV therapy, and suggest areas for future investigation.

## 2. Placing NK Cell Biology in the OV Context

Although a corpus of evidence has delineated both the role of NK cells in tumor clearance for various tumor models and their role in viral eradication, the importance of NK cells in response to OV therapy is just beginning to be appreciated. In order to decipher their role in the OV context, it is important to first understand the basic properties of NK cell biology. Human NK cells are divided into separate CD56^bright^ and CD56^dim^ populations that differ in their functional capacity and localization [10]. Approximately 90% of circulating and splenic NK cells are CD56^dim^CD16^+^, express perforin, and possess cytotoxic capacity when interacting with target cells [11]. In contrast, CD56^bright^CD16-NK cells are detected in lymph nodes and tonsils, lack perforin, and readily produce cytokines such as IFN-*γ* in response to stimulation with IL-12, -15, and -18 [12, 13]. In mice, NK cells have been differentiated into three subsets according to CD11b and CD27 expression [14]. NK cell differentiation in mice occurs from a relatively immature C11b^dull^CD27^+^ state to the double positive CD11b^+^CD27^+^ and ultimately to the senescent CD11b^+^CD27^dull^. Notably, both the double positive and senescent NK cells have been demonstrated to secrete IFN-*γ* and carry out cell-mediated cytotoxicity. While investigators have identified differential anatomical distribution for each type of NK cell in wild type mice, there have been no examinations into the maturation state of NK cells within the tumor microenvironment either in the absence or presence of OV. Despite the differences in developmental and activation markers on NK cells in mice and humans [15], a basic understanding of the role of NK cells in response to OV therapy in mice establishes a framework for future studies in clinical trials.

While certain NK cells are sufficiently mature to produce both cytotoxic and cytokine responses, these two functions are products of the cytokine microenvironment. For instance, type I interferon, IL-12, and IL-18 are critical for the induction of NK activation [16]. Moreover, much like T cells require “priming” for full activation, IL-15 has been elucidated as a cytokine that is critical for the priming function of murine NK cells [15, 17]. While initial efforts have examined the role of certain activating cytokines within the tumor microenvironment following OV therapy [18, 19], additional work is needed to understand their roles in OV clearance and tumor killing.

**Figure 2:**
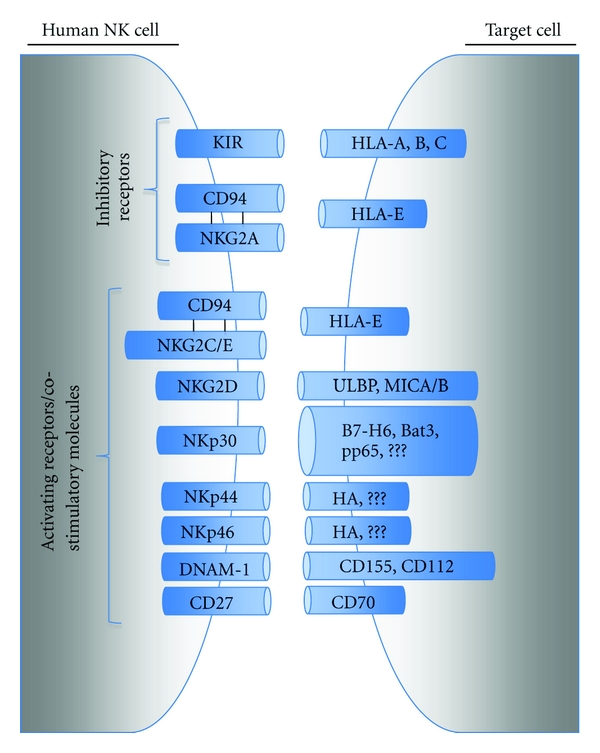
NK cell activation is mediated by a variety of cell surface inhibitory and activating receptors that recognize cell surface ligands on target cells. Viral infection, oncogenic transformation, and cellular stressors result in the downregulation in ligands for NK inhibitory receptors while concomitantly increasing the expression of NK activating ligands. Despite the presence of both activating and inhibitory signals on target cells, the overall balance of these signals dictates NK activation and target cell clearance.

NK cells are able to carry out their diverse repertoire of activities through a detection system that relies on the engagement of a variety of cell surface activating and inhibitory receptors on NK cells (Figure 2). Through the binding of these receptors, a dynamic equilibrium is achieved that differentiates the recognition of  “self” cells from transformed target cells. The activating NK cell receptors detect the presence of ligands on cells that are in a “distressed” state. These include stress ligands in mice (e.g., RAE1, H60, and MULT1) and humans (e.g., ULBP1-3 and MICA/B) that bind to the NKG2D activating receptor on NK cells. Natural cytotoxicity receptors (NCRs) are a family of activating receptors critical for mediating NK killing. They consist of NKp46 which is endogenously expressed on NK cells and NKT-like cells in both mice and humans [20]; NKp44 which is solely expressed on human NK cells, with constitutive expression only after cytokine stimulation, and plasmacytoid dendritic cells [21]; NKp30 which is exclusively expressed on resting and activated human NK cells [22]. While the identity of ligands for NCRs is a field of intense investigation [23], recent discoveries have identified influenza hemagglutinin (HA) as an activating ligand for NKp46 and NKp44 [24]. Besides mediating the eradication of tumor and virally infected cells, NKp30 interacts with immature dendritic cells (imDCs). Following NKp30 binding to an unknown ligand on imDCs [25], the imDCs are subsequently either killed or develop into mature DCs that can mediate a Th1 response [26] culminating in tumor/viral eradication. To counteract this process, human cytomegalovirus tegument protein pp65 impedes NKp30 activation through NKp30-CD3*ζ* receptor dissociation and the concomitant circumvention of NKp30-mediated maturation of dendritic cells [27, 28]. Known activating ligands for NKp30 include two activating cellular proteins. These include B7-H6 [29], a cell surface protein associated with tumor formation and Bat3 [30], a released cellular stress protein. In addition to their activating receptors, NK cells also possess an array of inhibitory receptors that are used to survey cells for the absence of constitutively expressed self-ligands. For instance, MHC-I expression is recognized by the inhibitory receptor killer cell immunoglobulin-like receptors in humans, lectin-like Ly49 dimers in mice, and CD94-NKG2A heterodimer in both species [15].

Taken together, by identifying cells that have absent MHC-I expression and/or upregulation of stress or virally encoded ligands for NK activating receptors, a target cell can be susceptible to NK-mediated lysis. By characterizing the NK ligand signature following OV infection and deciphering the receptor-ligand interactions that are responsible for NK recognition of virally infected cells, a mechanistic understanding can potentially guide the development of therapeutic approaches to selectively target the NK receptors implicated in either the anti-OV or antitumor response.

## 3. The Host Response to OV Therapy

The rapid response of innate immunity, consisting of NK cells, monocytes, macrophages, and neutrophils provides an initial and potent line of defense for the host and limits initial viral infection, replication, and spread; facilitates the maturation of antigen presenting cells; communicates with the adaptive arm of the immune system to regulate its response. Due to the rapid decline in viral titers within days of inoculating various oncolytic viruses [31, 32], the innate immune response has been implicated as a critical factor in this response. While neutrophils are the first antiviral responders that are recruited to a site of infection, efficient viral clearance at the cellular level requires both NK cells and monocyte-derived cells. Activated NK cells mediate direct lysis of infected target cells by releasing cytotoxic granules containing lytic enzymes [33] or by binding to apoptosis-inducing receptors on target cells [34]. NK cell-mediated preferential lysis of HSV- or vaccinia-virus-infected cells has been shown to prevent viral dissemination to neighboring cells [35]. While recruitment of NK cells to infected tumor tissue has correlated with reduced viral spread and OV efficacy, IFN-*γ* production by NK cells has also been shown to set the stage for the subsequent adaptive immune response [36, 37].

**Figure 3:**
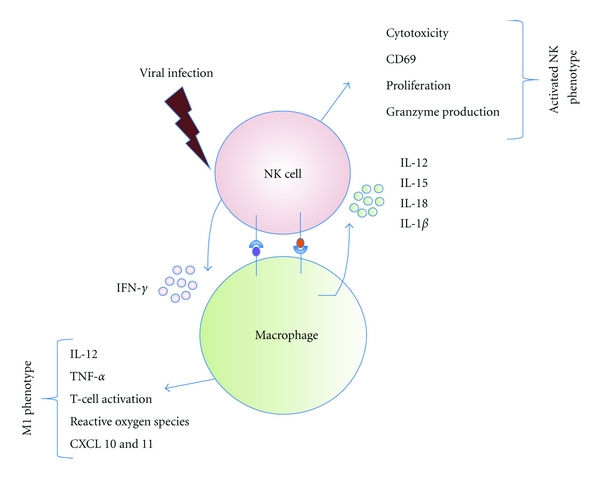
Innate immune effector cells, including NK cells and macrophages, represent an initial barrier to viral infection, replication, and spread. Following infection, NK cells are recruited to the site of infection and adopt an activated phenotype. Through their IFN-*γ* production, they also facilitate the maturation and activation of macrophages which adopt an inflammatory “M1” phenotype. Lastly, macrophages create a feedback loop by producing a variety of inflammatory cytokines that mediate NK activation. This inflammatory response creates a potent antiviral microenvironment while also communicating with the adaptive immunity.

Apart from NK cells, macrophages also play a critical role in OV clearance. Upon viral infection, resident or recruited macrophages initially secrete IL-12 to activate NK cells while NK cells complete the feedback loop by secreting IFN-*γ*—the prototypic macrophage activator, without which macrophages cannot clear microbes [36] (Figure 3). In fact, recruitment of infiltrating monocytic cells has been shown to coincide with clearance of over 80% of HSV-derived oncolytic viral particles [38, 39]. Increased intra-tumoral presence of macrophage/microglia cells has also been reported in human patients treated with adenovirus [40, 41] or HSV-1-derived OV [42] indicating the global significance of macrophages in OV therapy.

Despite their antiviral properties, NK cells have pleiotropic effects that may also be critical in tumor killing. NK cells have been shown to augment the tumoricidal effects in various models. This includes the most well-studied example of a melanoma model in which NK cells have been defined as an essential cellular component for VSV efficacy [43]. In this model, NK cells functioned synergistically with the adaptive antitumor immune response, launched in response to viral antigens expressed by tumor cells. Therefore, it appears that NK cells can serve a dual function—both as potential inhibitors of viral replication and as critical mediators to establish an effective antitumor immunity following viral antigen presentation within the tumor cells. These findings emphasize the impact of variations in tumor models, anatomical location of the tumors, and properties of the viruses that are being tested [2].

In future studies, a refined approach will be needed to manipulate individual cell populations while considering both the timing and nature of the intervention in order to maximize therapeutic regimens. One promising, albeit simplistic, approach will be to combine OV inoculation with transient immunomodulation in order to achieve initial viral replication, followed by restoration of immune activity to harness the immunotherapeutic potential of virotherapy.

## 4. NK Deficiencies

NK cell deficiencies, while being a rare phenomenon, provide valuable information about the role of these cells in antimicrobial defense and potentially the NK response to OV. While these deficiencies are relatively uncommon, they indicate the essential nature of these cells in host defense. The most informative group of disorders involves an isolated human NK cell deficiency that is associated with a specific gene mutation [4]. The only known human gene alteration resulting in an isolated NK cell deficiency results from a polymorphism in CD16, the IgG Fc receptor which is activated following binding to IgG. In this polymorphism, the CD16 epitope recognized by mAb B73.1 is changed by a T → A substitution at position 230 resulting in L48 → H [4]. Individuals homozygous for this alteration have phenotypically normal NK cells but are not recognized by mAb B73.1 [44, 45]. Several individuals have been documented to have a homozygous 48H phenotype and they reported to have recurrent viral infections. In particular, a 5-year-old girl was documented to have frequent respiratory infections, recurrent HSV stomatitis, and recurrent herpetic whitlow [45]. This child was deficient in NK cell cytotoxicity against K562 target cells but had normal antibody-dependent cellular cytotoxicity (ADCC). Taken together, this 48H phenotype suggests the importance of this epitope in resistance to viral infections.

A second group of NK cell deficiencies result from unknown gene mutations. The most striking example of human NK cell deficiency is from a female adolescent with an absolute NK cell deficiency based on a lack of lymphocytes expressing CD56 or CD16 and an absence of both NK cell cytotoxicity and ADCC. This patient presented with disseminated, life-threatening varicella infection and subsequently developed both CMV pneumonitis and cutaneous HSV [5]. There have also been reports of individuals with functional NK deficiency in which NK cells are present as a normal percentage of peripheral blood lymphocytes, but are deficient in activity. For example, four patients have been reported with widespread or invasive HSV disease, all with basal NK cell cytotoxicity against HSV-infected fibroblasts [46].

Although isolated NK cell deficiencies present the opportunity to understand NK cell specific genes and NK cell roles in human antimicrobial defense, a variety of other diseases have NK cell deficiency as a component. For instance, in Griscelli syndrome patients have variable immune deficiencies that typically include a marked reduction in NK cell cytotoxicity but an ability to induce cytotoxicity upon IFN-*α* or IL-2 stimulation [47–49]. Despite this variable responsiveness, patients with this syndrome have a propensity for EBV and HSV infections [47]. In leukocyte adhesion deficiency (LAD), patients have elevated peripheral leukocytes and in some patients, a corresponding recurrence of HSV infection [50, 51]. In these patients, the ability of NK cells to mediate cytotoxicity, ADCC, or kill HSV-infected target cells is severely attenuated. In LAD, there are a variety of mutations in the *β*2 integrin CD18 [52]. This results in the inappropriate expression of various key adhesion complexes including LFA-1 (CD11a/DC18) and Mac-1 (CD11b/CD18). Notably, LFA-1 associates with the immunoglobulin-like activating receptor DNAM-1 [53]. Taken collectively, the findings from both isolated human NK cell defects and diseases that include NK cell deficiencies suggest that human NK cell activity is especially important in limiting viral infection and may similarly attenuate oncolytic viral propagation.

## 5. The Interface between NK Cells and Oncolytic Viral Therapy

### 5.1. NK Cells and Antitumor Immunity

In fully immunocompetent animal models, the variables that ultimately determine clinical success are the amount of viral replication inside the tumor, the antiviral immune response elicited by viral infection, and the stimulation of an antitumor immune response. However, differences in oncolytic viral vectors, tumor models, and the anatomical locations of tumors add a layer of complexity that makes broad conclusions about host immunity difficult to achieve. Innate immune responses have the potential of mediating cytotoxicity directly against tumors while simultaneously mediating downstream immune response [54]. Among the innate immune cell compartment that mediates this response, NK cells stand out as a key cellular factor [55]. While the presence of NK cells within human tumors is associated with a positive prognosis [6–9], their infiltration within many tumors is often sparse [56, 57]. Since NK cells possess both antiviral and antitumor properties, it is not surprising that their involvement is equally controversial. While there are examples of studies that have found no involvement of NK cells in response to oncolytic viral infections [58, 59], the majority of studies find that they are relevant in some capacity. To start, we will focus on studies that both highlight the need of achieving antitumor immunity and determine the essential role of NK cells in mediating this response.

One of the first reports to mechanistically describe the essential nature of antitumor immunity was the work of Diaz et al. in which VSV was administered intratumorally for the treatment of an immunocompetent B16 melanoma model [43]. In this study, depletion experiments were performed to demonstrate that tumor regression was achieved in a CD8 and NK cell-dependent manner. While markers of NK cells and CD8 T-cell activation were not extensively examined, the authors did observe that CD8 priming correlated with increased cell counts in both the tumor and draining lymph nodes; however, NK cell numbers remained unchanged following infection. Corroborating the relevance of these findings, the treatment of prostate cancer with reovirus overrode the prominent immunosuppressant milieu of prostate adenocarcinoma [60, 61] and elicited an antitumor CD8 T-cell response along with prominent NK cell infiltration [62]. Miller and Fraser also found that intratumoral therapy with oHSV for metastatic melanoma was abrogated in syngeneic models lacking NK and T-cell subsets [63]. Lastly, inoculation of HSV-1716 induced the production of IFN-*γ* inducible chemokines from human DCs along with the migration of NK and CD8 cells into murine tumors [64]. Taken collectively, these findings from various tumor models treated with VSV, reovirus, and oHSV highlight the apparent relevance of NK and T cells as mediators of antitumor efficacy [2].

Interestingly, a recent Phase I trial examining intravenous administration of oncolytic reovirus found that CD8 and NK cells increased by 33% and 38%, respectively, following OV infection [65]. These findings appear to highlight the relevance of these cellular components in the clinical setting. Despite these observations, increases in immune cell numbers do not necessarily correlate with immune cells activation in response to infection. As a result, future studies are needed to evaluate the phenotypic profile and relevance of each immune cell component following oncolytic viral infection.

In addition to the CD8 and NK response, Diaz et al. examined the significance of regulatory T cells (Tregs) in their model [43]. Following viral inoculation, increased numbers of Tregs were detected within the tumor. Surprisingly, Treg depletion did not increase antitumor efficacy by relieving the suppression of antitumor CD8 cells; rather, the antiviral immune response was significantly enhanced following Treg depletion, resulting in both decreased viral titers and decreased OV efficacy. In this model, these findings suggest that Treg suppression is active at the level of antiviral, rather than antitumor immunity.

The treatment of B16 subcutaneous tumors and lung metastasis can also be treated with intravenous VSV, albeit using a different mechanism. In the study by Kottke et al., vascular leak syndrome (VLS) correlated with enhanced oncolytic VSV localization to subcutaneous and metastatic lung tumors [66]. VLS is induced following IL-2-mediated endothelial cell injury which was exacerbated with Treg depletion [67, 68]. The authors hypothesized that this endothelial damage and concomitant vascular permeability created an environment that facilitated viral access from the circulation to the tumor. Interestingly, NK cells were critical for VLS-mediated localization and spread through the tumor while also allowing for the continued delivery of virus to tumors in the presence of previously vaccinated mice [66].

In contrast to the work by Diaz et al. [43], the induction of VLS through Treg depletion induced a markedly different immune response to OV infection [66]. While IL-2 expands the pool of NK cells *in vivo*, these cells are kept in check by Tregs which are similarly expanded by IL-2. Thus, depletion of Tregs appears to result in hyperactivated NK cells manifesting in enhanced VLS, cytokine production, and cytotoxic effector functions [66]. Moreover, this efficacious response was recapitulated in the same tumor model using i.v. administration of reovirus combined with IL-2 and cyclophosphamide (CPA) [69]. When given at low doses, CPA has been demonstrated to enhance immune responses against tumors through transient depletion of Tregs [70, 71]. Consistent with the findings with VSV combined with IL-2 and Treg depletion [66], reovirus/IL-2/CPA cotherapy achieved an activated NK phenotype that achieved significant tumor regression [69].

Somewhat surprisingly, however, this state of NK cell hyperactivation, which presumably includes antiviral properties, correlates with increased VSV localization, replication, and spread. These findings suggest that in a VLS virotherapy model, the antiviral properties of hyperactivated NK cells are overridden by their ability to localize systemically injected virus to the tumor. These findings were also in contrast to the findings of Diaz et al. [43] in which Treg depletion was detrimental for CD8/NK cell-mediated efficacy of IT administration of VSV. Thus, Treg depletion clearly has positive and negative outcomes and emphasizes the need to consider opposing interactions between antitumor and antiviral immune cells.

While the field of oncolytic viral therapy is built upon the premise that a small initial inoculum will go through progressive rounds of viral replication in order to achieve tumor clearance, the work of Galivo et al. used a mutant form of VSV to modify this fundamental principle [72]. Rather than just relying on viral oncolysis, it is becoming increasingly apparent that antitumor immunity is a critical factor for VSV-mediated efficacy. This was clarified by Galivo et al. in which a single replication cycle VSV vector, but not a replication defective or UV inactivated virus, was found to achieve equal therapeutic efficacy compared to a fully replication competent VSV vector [72]. Thus, the ability of oncolytic VSV to proceed through multiple rounds of viral replication is unnecessary; rather, the immune response to intratumoral injection of a live, viable virus that is able to express its genome is the essential effector mechanism for tumor clearance [72]. Further supporting this premise, both viruses elicited a nearly identical NK and CD8 T-cell immune response, thereby confirming that antiviral immunity and the ability to elicit an acute proinflammatory response is an essential component for achieving antitumor efficacy that is seen in the B16 model [72].

An additional mechanistic component for VSV-mediated clearance of B16 is the role of IL-28 and NK cells in this process. In the study by Wongthida et al., IL-28 expression following VSV infection is identified as a key mediator of antitumor immunity [73]. Both the expression of IL-28 and the presence of its cognate receptor on B16 tumors were required for therapeutic efficacy. Moreover, IL-28-mediated activation of bone marrow cells to induce bystander cytotoxicity against B16 while maintaining an environment conducive for viral replication and spread [73]. Although IL-28 was produced from GR1^+^ and Macs3^+^ and not NK cells, depletion of NK1.1 cells eliminated cytotoxicity against B16 cells. It appears that this is coordinated through IL-28 induction of NK ligands on B16 cells and IL-28-mediated activation of IFN-*γ* production from NK cells (Table 1). Thus, IL-28 was identified as a novel mediator of NK cell activation that is essential for VSV therapeutic efficacy. Further work is needed to extrapolate these findings to other tumor models and oncolytic viral vectors; however, this initial report suggests that screening tumors for IL-28 receptor may represent a useful prognostic marker for predicting therapeutic response [73].

A recent study by Granot et al. has added additional evidence to the relevancy of NK cells in mediating viral mediated tumor clearance. Using a replication deficient Sindbis virus to treat SCID mice bearing ES-2 ovarian carcinoma xenografts, the authors found that tumor clearance was dependent upon the presence of functional NK cells [74]. In addition, the efficacy of a recombinant Sindbis/IL-2 expressing virus was dependent upon NK cells and IFN-*γ* production that ultimately mediated macrophage polarization towards an inflammatory M1 phenotype. These findings highlight the role of NK-mediated tumor clearance in immunodeficient mice treated with a replication defective viruses. It is important to interpret these findings in the context that viral replication was not a goal with this viral vector. Moreover, it is important to note that NK cells were the key antitumor mediator in the SCID mice used in the experiment. As a result, future work will need to discriminate (a) whether NK cell activity is still essential when viral replication is desired, and (b) the relative importance of NK cell coordinated adaptive antitumor immunity in immunocompetent models.

### 5.2. Pharmacologic Modulation of the Host Response to OV

While antitumor immunity can be a critical tool for OV efficacy, various pharmacologic cotherapies have also been used to counteract this response and synergize with OV therapy. For instance, the complement system is rapidly activated following viral infection and is intended to directly neutralize virus; consequently, abrogating this response could potentially enhance OV efficacy. This question was addressed by using cobra venom factor which depletes the C3 component of the complement system and was subsequently shown to enhance OV infection [82].

Cyclophosphamide (CPA) has also been used to attenuate antibody-mediated activation of the complement system, serum neutralization of virus, and reduce peripheral blood mononuclear counts that are responsible for producing an antiviral cytokine response [19]. Due to the pleiotropic immunosuppressive nature of CPA and its ability to halt the antiviral immune response, *in vivo* CPA treatment significantly reduced viral clearance and increased viral propagation while reducing immune cell filtration [19, 83]. Taken together, these findings suggest that targeting the immune response to OV therapy is a particularly useful modality towards achieving enhanced OV efficacy.

While angiogenesis provides resources to growing tumors, this increased vascularity is also associated with increased immune cell trafficking. As a result, antiangiogenic agents have the potential of reducing not only tumor growth but also the antiviral tumor microenvironment. For example, cilengitide is an antiangiogenic, cyclic RGD (cRGD) peptide that was originally identified as antagonists for the integrins *α*v*β*3 and *α*v*β*5 [84]. cRGD also limits leukocyte recruitment to sites of inflammation [85], reduces myeloid cell adhesion and transendothelial cell migration [86, 87]. When combined with OV, cRGD limited both OV-mediated inflammatory gene expression and CD45 leukocyte recruitment [18]. This reduced inflammatory response resulted in increased OV propagation *in vivo* and significantly enhanced therapeutic efficacy of OV in animals with intracranial tumors [18].

An additional pharmacologic approach that has been demonstrated to enhance OV therapy is the use of the histone deacetylase inhibitor valproic acid (VPA). VPA has been shown to enhance the efficacy of oncolytic HSV [88] through the inhibition of IFN-I, STAT-1, PKR, and PML signaling within infected glioblastoma cells. By targeting intracellular mediators that are responsible for creating an antiviral state [89], this therapeutic approach targets the antiviral host response prior to the recruitment of antiviral cellular mediators.

### 5.3. NK Cells and Antiviral Immunity

Despite a number of studies suggesting that natural killer cells are a critical component for achieving tumor clearance following oncolytic viral therapy, the deleterious nature of innate immunity has also been well documented with immunosuppressive cotherapies that enhance OV efficacy. Mathematical modeling has previously shown that the timing of the antiviral innate immune response to OV can be detrimental to therapy [90]. A variety of important changes associated with antiviral immunity occur in the tumor microenvironment in response to oHSV treatment of malignant GBM. These include profound increases in IFN-*γ* transcript and protein levels; upregulation of IFN-*γ* inducible chemokines; increased hyperpermeability with an associated inflammatory cell infiltrate; a rapid rise in an inflammatory transcriptome including type I interferon, TNF*α*, iNOS, and IL-15 [18, 32, 83, 91].

Studies in multiple tumor models and oncolytic viruses have observed a rapid decline in viral titers that occurs within days of inoculating various oncolytic viruses [32], suggesting that impediments are in place that limits successive rounds of viral replication. Using a GBM model, the clearance of over 80% of oHSV occurs in an IFN-*γ* dependent manner and corresponds with the rapid recruitment of NK cells and peripheral macrophages into the site of viral infection, suggesting that this response is a potential factor mediating oHSV clearance [64]. Moreover, transient immunomodulation with cyclophosphamide attenuated NK cell and macrophage recruitment to the site of oncolytic HSV-1 infection while resulting in profoundly increased viral titers and tumor clearance. Apart from HSV-1-derived OV, CPA has also been shown to increase the oncolytic capacity of other OVs derived from HSV-2 [92], adenovirus [39], and reovirus [69, 93]. In fact, based on the promising preclinical results seen with CPA and OV, the combination of CPA with measles virus is currently being evaluated for safety and efficacy in human patients [94]. Similarly, clinical trials of reovirus with CPA (clinicatrials.gov, NCT01240538) and adenovirus with CPA (A. Hemminki, personal communication) are being conducted.

The finding that CPA enhances OV therapy through the suppression of immune cell recruitment was validated through a study demonstrating that macrophage depletion enhanced the efficacy of oHSV therapy in glioblastoma [91]. Clodronate encapsulated in liposomes is engulfed by phagocytic cells resulting in intracellular accumulation of apoptosis inducing clodronate [95]. When combined with OV therapy, CL-mediated depletion of peripheral phagocytic cells resulted in a 5-fold increase in OV titers in intracranial glioblastoma. While these findings partly recapitulated the effect of CPA on OV replication, they were unable to achieve enhanced *in vivo* survival demonstrated with CPA [91]. A potential reason for these findings may relate to the inability of clodronate to cross the blood brain barrier, thereby limiting its ability to deplete phagocytic microglial cells in addition to peripheral macrophages. Alternatively, macrophages may represent just one of multiple barriers present within the innate immune response to OV. In fact, a study by Breitbach et al. found that neutrophil depletion increased oncolytic viral titers and enhanced tumor clearance [96].

These findings suggest that OV replication may be limited by cellular components of the innate immune system shortly after viral infection. While we have previously demonstrated that microglia and macrophages play a critical role in limiting the therapeutic efficacy of OV [91], the role of NK cells has received less attention. In a series of two separate studies, Altomonte et al. detected NK cell infiltration into hepatocellular tumors treated with VSV and demonstrated a critical role for NK cell-mediated viral clearance in this model. First, intratumoral VSV replication and tumor killing was significantly enhanced in mice depleted of NK cells [97]. Second, a recombinant VSV encoding a viral chemokine binding protein (equine herpesvirus-1 glycoprotein G) attenuated NK cell recruitment, enhanced viral titers and tumor necrosis, and dramatically increased the overall survival of tumor-bearing mice compared to mice treated by parental VSV [97]. Due to the broad range of binding partners of the chemokine binding protein, an additional recombinant VSV encoding UL141 was created in order to more specifically inhibit NK cell recruitment and activation [77] (Table 1). UL141 is derived from human CMV that inhibits NK cell activation by blocking cell surface expression of CD155 on infected cells, thereby attenuating DNAM-1 mediating signaling on NK cells. In this study, rVSV-UL141 similarly enhanced virotherapy by specifically targeting the NK response to viral infection [77]. These series of findings suggest that NK cell recruitment and activation, at least in some models, can be detrimental to viral oncolysis and should be circumvented.

Based on findings that oHSV elicits brisk NK recruitment to the site of infection and CPA modulation of innate immunity enhances oHSV-mediated glioblastoma killing [18, 19, 32, 83, 90, 98], the functional relevance of these cells *in vivo* brain tumor models remains unclear and additional studies will be needed to determine this. The most recent clinical trial for oncolytic HSV in recurrent glioblastoma patients attempted to determine evidence of HSV replication and the extent of immune infiltration into the tumor microenvironment following OV administration [42]. While viral replication was observed in a portion of the patients enrolled in the study, there was significant variability in viral levels between patients. These findings are in contrast to the assumption in the field of OV therapy that even small initial inoculums should amplify significantly following successive rounds of viral replication [91, 99–101]. Additionally, immunohistochemical (IHC) analysis of tumor tissue demonstrated that G207 administration elicited a robust increase in CD3, CD8, CD20, and HAM56 staining. Additionally, testing of the genetically engineered mutant adenovirus (ONYX-015) in a phase I trial for glioblastoma provided similar results. Notably, when biopsies were taken from recurrent tumors, a significant inflammatory, mononuclear infiltrate was observed within the tumor microenvironment. Notably, in both of these studies, staining with the pan-NK maker CD56 was not included. These collective IHC findings are consistent with our preclinical findings [18, 32] and suggest that OV infection/replication causes the recruitment of inflammatory infiltrates into the site of viral infection. The nature of this histological feature is uncertain. For example, it is unclear whether these findings correlate with a potential benefit by stimulating an antitumor immune response or a detriment by eliminating OV initially after infection thereby preventing initial rounds of viral replication within the tumor. Taken together, the findings from these clinical trials demonstrate the need for a clearer understanding of the host-based factors and cellular mediators that are responsible for limiting viral infection, replication, and spread. By clarifying the host response to the virus, subsequent clinical trials can be designed to modulate these obstacles to viral propagation and achieve enhanced OV efficacy.

### 5.4. NK Cell Interactions with OV Infected Cells

Despite the conflicting viewpoints that NK cells are either a benefit or a hindrance to OV efficacy, most investigators would argue that NK cells and their place within innate immunity have a critical role in achieving success with this therapeutic modality [2]. As a result, the knowledge gained from uncovering the mechanistic signals governing NK-mediated recognition of OV infected cells has the potential of benefiting both schools of thought. For instance, in cases where the NK response is deleterious to OV efficacy, it may be necessary to design oncolytic viruses that express decoys or suppressors of NK activating ligands or combine the oncolytic virus with cotherapies that achieve similar NK avoidance of oHSV infected target cells. In tumors where NK-mediated killing is beneficial to OV therapy, the opposite approach could be used so that the oncolytic virus is combined with an immunostimulatory agent that heightens the expression of critical NK activating ligands. In either case, it will be necessary to uncover the underlying signals that mediate NK recognition of OV infected tumor cells.

NK cells are able to carry out their diverse repertoire of activities through a detection system that relies on the engagement of a variety of cell surface activating and inhibitory receptors on NK cells that bind MHC class I and class I-like molecules (Figure 2). Through the binding of these receptors, a dynamic equilibrium is achieved that differentiates the recognition of  “self” cells from malignantly or virally transformed target cells. While a number of viral vectors are being tested for the treatment of an equally diverse array of tumors, studying the NK response to oHSV infection of GBM, for example, can be extended to various virotherapy models. GBMs are readily killed by NK cells *in vitro *[102], and despite an intense immunosuppressive tumor microenvironment as the GBM progresses, a recent report found that peripheral NK numbers were not altered in patients with GBM [103]. NK cells have also been demonstrated to preferentially lyse virally infected target cells through either the elaboration of cytotoxic granules containing lytic enzymes or through the binding of apoptosis-inducing receptors on target cells [33]. Through the expression of both virally and tumor/stress associated ligands for NK activating receptors, a target cell such as an infected GBM can become susceptible to NK-mediated lysis. Countering this is the production of TGF-*β* and related molecules by the refractory GBM cells that impede both NK cell production of IFN-*γ* [104] and the ability of NK cells to directly lyse tumor cell targets [105].

Recent discoveries have explored the role of HSV-1 infection in modulating NK activating ligand expression. HSV-1 infection of fibroblast cells, through its immediate early gene product ICP0, resulted in increased susceptibility to NK-mediated lysis in an MHC-I independent fashion [79]. NK activation was achieved independently of the NK activating receptor NKG2D [79]. Rather, NK stimulation was elicited through the expression of an unknown ligand(s) of the NCRs on the cell surface of infected cells. Moreover, HSV infection was subsequently found to downmodulate NKG2D ligand expression due to late viral gene products [78] (Table 1). These findings demonstrate the modulation of ligands for NK activating receptors following wild type HSV infection; however, neither the identity of these ligands nor the nature of their cooperative binding to NK activating receptors is currently resolved.

Beyond cell-mediated viral clearance, NK cells are potent producers of IFN-*γ* [104]. In the context of oHSV therapy, the detrimental role of this antiviral cytokine has been uncovered through an IFN-*γ* depletion study that resulted in enhanced intratumoral viral titers [32]. Additionally, IFN-*γ* is vitally important in the activation of macrophages, thereby facilitating their ability to kill both viral and obligate intracellular pathogens [106]. Collectively, these findings demonstrate the critical nature of NK cells in coordinating the antiviral response to oHSV therapy.

A number of groups have also studied the ability of human glioblastomas to induce NK cell cytotoxicity. Based on their expression profile of ligands for activating NK receptors [102, 107], NK cells have been demonstrated to actively lyse a variety of glioblastoma cells in a NKp46 and DNAM-1 dependent manner [102].

The mechanism of NK-mediated cytotoxicity in other oncolytic viral models is also being explored. Recently, work by Bhat et al. found that oncolytic parvovirus infection of PDAC cell lines resulted in increased IFN-*γ*, TNF*α*, and MIP1a/b release from NK cells in coculture [80]. Moreover, parvovirus infection appeared to sensitize virally infected tumors cells to NK-mediated killing through the downregulation of MHC-I, enhanced expression of NK activating ligands such as CD155, and involvement of multiple NK activating receptors including NCRs, DNAM-1, and NKG2D [80]. Based on the findings that target cells infected with wild type influenza A virus (IAV) were exquisitely sensitive to NK-mediated lysis through NKp46 recognition of HA expressed on virally infected targets, an oncolytic IAV was developed. This novel virus lacks the NS1 gene and grows efficiently in IFN-resistant malignant cells with concomitant Ras overexpression [108]. Using the ΔNS1 IAV infected prostate cancer cells, Ogbomo et al. found that NK cells overrode MHC-I inhibition and exhibited rapid ERK activation, increased degranulation, and heightened NKp46-mediated target cell lysis compared to uninfected cells [81]. These findings suggest that second-generation versions of these viruses that coexpress an NK activating cytokine such as IL-2 or IL-12 may be a useful tool in activating NK cells *in vivo*, particularly in cases where the immunosuppressive environment of the tumor results in resistance to NK-mediated lysis.

By deciphering the critical receptor-ligand interactions that are responsible for NK activation following viral infection, this has the potential of paving the way for the development of novel therapeutic approaches. This can be achieved by either the selective blockade of NK receptors implicated in the anti-OV response or the development of ways to stimulate the critical receptor-ligand interactions that are essential for downstream immunotherapy.

## 6. Future Perspectives

Nearly two decades after the first published report of oncolytic viral therapy [40], investigators using these viruses have made remarkable progress in their preclinical testing and evaluation in clinical trials. Moreover, the recent approval of the H101 oncolytic adenovirus in China [109], the numerous clinical trials in place within the United States and Europe, and the acquisition of BioVex by Amgen suggest that virotherapy will gradually enter the armamentarium of tomorrow's physicians. In order to achieve widespread clinical applicability, however, certain obstacles must be overcome. For instance, infected cells have various antiviral defense mechanisms that must be circumvented to achieve sustained viral replication; however, circumventing this response must be countered with concerns about uncontrolled viral replication and toxicity. Additionally, host immunity, particularly innate immunity, is a first line of defense against foreign pathogens that has been demonstrated to impede virotherapy; however, immune suppression potentially impedes the antitumor immune response that has been shown to synergize with viral oncolysis. Lastly, coadministration of pharmacological agents that cooperate with viral mediated tumor clearance shows significant promise; however, the comparative effectiveness of treating various tumors with the appropriate virus/drug combination must be thoroughly evaluated in clinical trials.

In recent years, significant attention has been directed towards the host immune response to oncolytic viruses. In particular, the role of initial immune responder cells, including NK cells, macrophages, and neutrophils, has been questioned. With their antiviral and antitumor properties combined with their ability to mediate macrophage activation, the NK cell response to virotherapy has elicited significant attention.

Despite recent progress, there are certain challenges that should be addressed in order to expand our knowledge of the NK response to OV therapy. To date, limitations in mouse strain susceptibility to certain oncolytic viral vectors have impeded the ability to evaluate the host immune response in fully immunocompetent animal models [110, 111]. Thus, there is an increasing need to study the NK response to OV in syngeneic, orthotopic tumor models that include the presence of tumor initiating cells and recapitulate the immunosuppressive tumor microenvironment that is frequently found in human cancers.

In order to fully investigate the role of NK cells following OV infection, future studies should also attempt to test OV in a syngeneic mouse model with specific NK deficiencies. By testing for OV efficacy and downstream immune cell activation, including macrophage and T-cell polarization, in each of these groups, it would be possible to delineate the critical NK components in antiviral and antitumor immunity to OV. Additionally, NKp46 is the only NCR present on murine NK cells. As a result, we are limited in our ability to test the significance of NKp44 and NKp30 against OV *in vivo*. To circumvent this problem, it would be possible to evaluate OV in the context of a humanized mouse model [112] where NKp46, NKp44, and NKp30 are expressed.

While a variety of cotherapies have been shown co-operate with viral oncolysis through immune cell suppression, additional work can be done to fine-tune this approach. For instance, it is shortsighted to think that antitumor immunity has no part in viral oncolysis. Rather, future work must identify the delicate balance between an initial suppression of antiviral immunity that facilitates initial rounds of viral replication and a downstream stimulation of antitumor immunity against tumor or viral antigens. This could potentially take the form of targeting NK cell depletion in a temporal manner whereby NK cells are attenuated initially after viral infection and then allowed to repopulate the site of infection a few days after viral inoculation. This later response would take advantage of their antitumor properties, macrophage activating properties, and their ability to induce an antitumor T-cell response.

An additional approach could focus on M1/M2 macrophage polarization following OV infection. Clinical reports have confirmed that tumors, such as glioblastoma, are typically associated with generalized immunosuppression, TGF-*β* production, and an M2 macrophage phenotype [103]. oHSV inoculation elicits an M1 macrophage response that is detrimental to OV efficacy [91]. Taken together, future studies could attempt to discern whether a temporary maintenance of the M2 phenotype initially after infection followed by a switch towards an inflammatory M1 state is effective at initially enhancing viral titers while eliciting antitumor immunity at later timepoints.

The role of NK receptor-ligand interactions deserves further exploration. Future studies should investigate the identity of the NK receptor ligands in virally infected cells; whether the ligands are viral in origin or expressed following cellular stress; whether they are expressed following infection with other oncolytic viral vectors. The identifying of ligands expressed after OV infection will be useful on multiple fronts. First it could potentially be targeted to enhance viral efficacy. For instance, if NKp30 and NKp46 are key receptors mediating premature viral clearance, suppressing the expression of these ligands could be particularly advantageous. Targeted suppression could be achieved through either pharmacologic means or through the creation of a novel oHSV that expresses a decoy for NKp30/NKp46 or inhibits ligand presentation on the infected cell surface. Second, while NK killing of virally infected cells may be deleterious for viral replication, it represents a novel target for mediating antitumor immunity. As a result, in instances where NK-mediated antitumor immunity is deemed beneficial, such as later time points after infection once productive viral replication is established, eliciting NKp30/NKp46-mediated tumor killing could be pursued as a viable therapeutic option.

With a number of oncolytic viral clinical trials in the pipeline, it will be critical for investigators to include the evaluation of NK cells in the immunological response to viral administration. Attention should be directed towards NK cell numbers in the periphery, within the tumor microenvironment and their distribution within the virally infected tumor. Moreover, the activation/developmental state of these NK cells should be evaluated. For instance, NK cells should be tested for CD56 and CD94 expression, whether the recruited/circulating NK cells are NCR^bright^ or NCR^dim^, and their functional capacity. By collecting this data from human samples, we can test the validity of our preclinical models and guide future experimental trials.

## Figures and Tables

**Table 1:** Relevant NK receptor-ligand interactions in the field of virotherapy.

Treatment	Altered NK ligand or receptor	Reference
IL-28	↑ RAE-1	[73]
	↑ H60	[73]
	↑ MULT-1	[73]

Valproic acid	↓ NKp30 and NKp46	[75]
	↑ MICA/B	[76]

VSV+UL141	↓ CD155	[77]

HSV	↓ MICA	[78]
	↑ NCR ligands	[79]

Parvovirus	↑ CD155	[80]

ΔNS1 influenza type A	↑ Influenza hemagglutinin	[81]
